# Discovering Host Genes Involved in the Infection by the *Tomato Yellow Leaf Curl Virus* Complex and in the Establishment of Resistance to the Virus Using *Tobacco Rattle Virus*-based Post Transcriptional Gene Silencing 

**DOI:** 10.3390/v5030998

**Published:** 2013-03-22

**Authors:** Henryk Czosnek, Assaf Eybishtz, Dagan Sade, Rena Gorovits, Iris Sobol, Eduardo Bejarano, Tábata Rosas-Díaz, Rosa Lozano-Durán

**Affiliations:** 1 Institute of Plant Sciences and Genetics in Agriculture, The Robert H. Smith Faculty of Agriculture, Food and Environment, The Hebrew University of Jerusalem, Rehovot 76100, Israel; E-mail: czosnek@agri.huji.ac.il; 2 Málaga-Consejo Superior de Investigaciones Científicas (IHSM-UMA-CSIC), Departamento Biología Celular, Genética y Fisiología, Universidad de Málaga, Campus Teatinos, Málaga, Spain; E-mail: edu_rodri@uma.es

**Keywords:** Tomato yellow leaf curl disease, geminiviruses, plant-resistance, tomato, VIGS, reverse genetics, plant-virus interaction

## Abstract

The development of high-throughput technologies allows for evaluating gene expression at the whole-genome level. Together with proteomic and metabolomic studies, these analyses have resulted in the identification of plant genes whose function or expression is altered as a consequence of pathogen attacks. Members of the *Tomato yellow leaf curl virus* (TYLCV) complex are among the most important pathogens impairing production of agricultural crops worldwide. To understand how these geminiviruses subjugate plant defenses, and to devise counter-measures, it is essential to identify the host genes affected by infection and to determine their role in susceptible and resistant plants. We have used a reverse genetics approach based on *Tobacco rattle virus*-induced gene silencing (TRV-VIGS) to uncover genes involved in viral infection of susceptible plants, and to identify genes underlying virus resistance. To identify host genes with a role in geminivirus infection, we have engineered a *Nicotiana benthamiana* line, coined 2IRGFP, which over-expresses GFP upon virus infection. With this system, we have achieved an accurate description of the dynamics of virus replication in space and time. Upon silencing selected *N. benthamiana* genes previously shown to be related to host response to geminivirus infection, we have identified eighteen genes involved in a wide array of cellular processes. Plant genes involved in geminivirus resistance were studied by comparing two tomato lines: one resistant (R), the other susceptible (S) to the virus. Sixty-nine genes preferentially expressed in R tomatoes were identified by screening cDNA libraries from infected and uninfected R and S genotypes. Out of the 25 genes studied so far, the silencing of five led to the total collapse of resistance, suggesting their involvement in the resistance gene network. This review of our results indicates that TRV-VIGS is an exquisite reverse genetics tool that may provide new insights into the molecular mechanisms underlying plant infection and resistance to infection by begomoviruses.

## 1. Introduction

Viral diseases threaten the production of agriculture plant crops. To establish a successful infection, viruses must hijack the cellular machinery and prevent or counteract the plant defenses. On the other hand, plants have developed a variety of resistance mechanisms, either ready to meet incoming pathogens or induced by them. High-throughput technologies allow following changes in gene expression upon virus infection at the genome level and evaluating the functions of these genes during infection [1,2], in susceptible as well as resistant plants [3]. Begomoviruses (genus *Begomovirus*, family *Geminiviridae*), a major virus family affecting agricultural crops worldwide, have been the subject of such studies [4,5,6]. Identifying the host genes selectively expressed during infection and determining their role is a pre-requisite to understand the process of begomovirus infection in susceptible and resistant plants. We review here how the use of a reverse genetics approach based on virus-induced gene silencing (VIGS) has allowed the identification of plant genes involved in infection and in resistance to begomoviruses of the *Tomato yellow leaf curl virus* (TYLCV) complex. 

## 2. Analysis of gene expression in plants using a reverse genetics approach based on virus-induced gene silencing

Plant innate response to virus invasion includes triggering resistance gene products, local cell death and systemic acquired resistance [7]. During the last decade, it appeared that RNA silencing is another, sequence-specific, universal plant defense mechanism against virus invasion [8]. It was discovered that replication of RNA and DNA viruses is associated with the accumulation of virus-derived small RNAs that help cleave viral messengers in a sequence specific manner [9,10]. This mode of RNA silencing was referred as post-transcriptional gene silencing (PTGS). Viruses encode suppressors of RNA silencing, which efficiently inhibit host antiviral responses [11]. RNA silencing of viruses led to the development of an outstanding reverse genetic tool now widely used in plant biology, known as virus-induced gene silencing (VIGS). In plants, VIGS is specifically targeted against the viral genome. However, with virus vectors carrying inserts derived from host genes, the process can be targeted against the corresponding mRNAs [12]. Hence, VIGS has emerged as an efficient tool to study gene silencing in plants [13]. 

One of the most common vectors currently used is based on the *Tobacco rattle virus* (TRV) [14,15]. This method uses a bipartite vector system designed between left and right borders of the *Agrobacterium* Ti plasmid. *TRVI* contains the RNA-dependant RNA polymerase (RdRp) and the MP components of the virus whereas *TRVII* contains multiple cloning sites (MCS) and the CP sequences. The bipartite plasmids are flanked by the 35S C*auliflower mosaic virus* promoter and a *Nopaline synthase* gene terminator. The MCS in *TRVII* allows ligation of DNA target sequences that will induce PTGS in the plant upon delivery by agroinoculation. The multiplication of the vector in the plant tissue triggers the cleavage of target sequence resulting in loss of expression [14]. Among other features, VIGS has been used to dissect the genetics of floral development and scent production [16], water deficit stress tolerance [17], embryogenesis, chlorophyll biosynthesis and disease resistance [18], and protective acyl sugars in trichomes [19]. The siRNAs-mediated RNA silencing has been exploited to engineer plants resistant to diseases by targeting the genome of viruses, viroids, insects and fungi [20]. 

TRV is not the only virus used as vector for PTGS studies. More than 30 viruses have been shown to have potential as VIGS vectors [21]. Among others, the tobamovirus *Tobacco mosaic virus* (TMV) and the potyvirus *Potato virus X* (PVX) have been engineered to target the plant phytoene desaturase gene (PDS), frequently used as a reporter gene for efficient silencing (the leaf loses its green color) [22]. The Hordeivirus Barley stripe mosaic virus (BSMV) served as vector to silence PDS, magnesium chelatase subunit H and plastid transketolase genes, and the powdery mildew resistance 5 gene PMR5 in Nicotiana benthamiana, barley and wheat [23]. Several geminiviruses have been engineered to serve as VIGS vectors. *Tomato golden mosaic virus* was used to silence the proliferating cell nuclear antigen (PCNA) and a subunit of magnesium chelatase in *N. benthamiana* [24]. Tomato leaf curl virus (ToLCV) served to silence tomato PCNA [25]. TYLCV was modified to serve as a gene silencing system in tomato and was applied to silence a viral silencing suppressor of Grapevine virus A (GVA), resulting in GVA-tolerant *N. benthamiana* plants [26]. *Cabbage leaf curl virus* (CaLCuV) was used to dissect the host geminivirus silencing mechanism in *Arabidopsis thaliana* [27]. The DNA1 satellite of the *Tobacco curly shoot virus* has been modified into a VIGS vector to study floral development [28]. *African cassava mosaic virus* (ACMV) was used to silence genes involved in glycoside synthesis in cassava [29]. Cotton leaf crumple virus (CLCrV) was used to silence a cotton magnesium chelatase subunit I gene [30]. 

## 3. Tomato yellow leaf curl viruses: a complex of begomoviruses infecting tomato plants worldwide

Tomato cultures (*Solanum lycopersicum)* worldwide are under the constant threat of diseases caused by geminiviruses belonging to the TYLCV complex [31]. In nature, the TYLCVs are exclusively transmitted by the whitefly *Bemisia tabaci* [32]. Members of the TYLCV complex have a single 2,700-2,800 nucleotide (n) circular ssDNA genome encapsidated in a geminate particle. The TYLCVs replicate in the nuclei of infected cells following a rolling-circle strategy, using a double stranded DNA intermediate replicative form as a template [33]. Their genome encodes two genes, V1 and V2; the complementary viral strand encodes four genes, C1 to C4. A 300 n intergenic region (IR) includes a stem-loop structure containing the origin of replication shared by all known begomoviruses and bidirectional promoters. V1 encodes the coat protein (CP); V2 encodes a movement protein (MP) and may also function as a silencing suppressor. C1 encodes a protein (Rep) necessary for replication, C2 a transcription activator (TrAP), C3 a replication enhancer (REn) and C4 a small protein embedded within the Rep that may act as a symptom determinant [34]. 

Plants have been genetically engineered to resist infection by members of the TYLCV complex. Strategies employed were based on expressing viral proteins, whether wild-type or mutants, of virus-binding proteins, and on viral gene silencing [35]. However, in view of the public reticence regarding genetically modified food crops, breeding remains a method of choice to obtain plants resistant to TYLCV [36]. Wild relatives of domesticated plant species constitute an invaluable reservoir of resistance genes, which have been tapped by plant breeders to improve agricultural crops [37]. It is thought that the expression of these resistances involves sets of genes that interact upon positive and negative signals within an interconnecting network [38]. Along domestication, these networks have been disrupted and resistances lost, probably because resistance alleles were linked with undesired horticultural qualities. Breeding has been instrumental in reconstituting (part of) the resistance gene network(s). 

Since the domesticated tomato *S. lycopersicum* is susceptible to TYLCV, breeders have introgressed resistance traits identified in wild tomato species (such as *S. chilense*, *S. peruvianum* and *S. habrochaites*) into *S. lycopersicum* [36,39]. As a result, the resistant tomato lines contain chromosomal fragments from the wild species on a domesticated tomato background, identifiable with polymorphic DNA markers [40]. Several loci from wild tomato species associated with resistance to TYLCV and related begomoviruses (coined Ty-1 to Ty-5) have been identified using such markers. The gene conferring TYLCV-resistance at the Ty-1 (from *S. chilense*) and Ty-5 (from *S. peruvianum*) loci have been identified (unpublished) but their function in the establishment of resistance is not known. 

## 4. Identification of host genes involved in TYLCV infection

### 4.1. A Nicotiana benthamiana system to monitor TYLCSV infection in combination with host gene silencing

**Figure 1:**
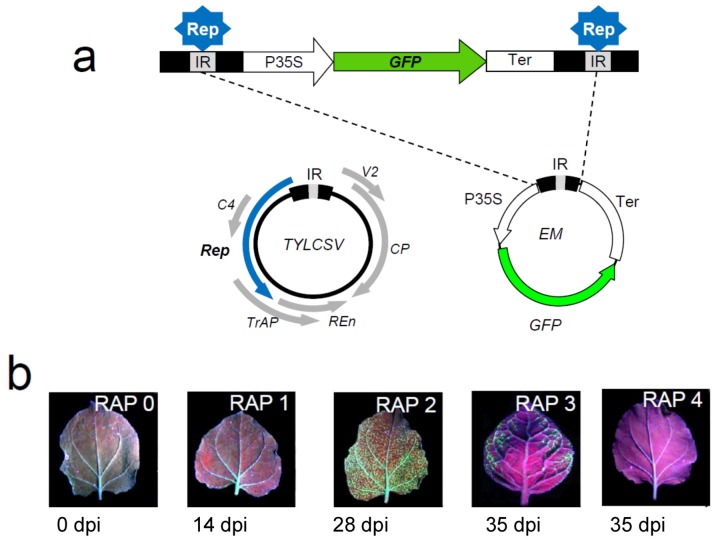
Generation and phenotypic analysis of TYLCSV-infected 2IRGFP *N. benthamiana* transgenic plants. **a. **Construct 2IRGFP contains a direct repeat of the TYLCSV IR encompassing a GFP expression cassette that contains the 35S CaMV promoter (P35S), the complete ORF of *GFP* and the NOS terminator (Ter). During TYLCSV infection, the viral Rep protein specifically recognizes the IRs flanking the cassette, and mGFP replicons are generated (EM), which in turn leads to a strong over-expression of the *GFP* transgene and the subsequent accumulation of the fluorescent protein. **b.** Evolution of virus replication-associated phenotype (RAP) in infected 2IRGFP plants at different days post-infection (dpi). A representative photograph of each RAP phenotype showing the extension and intensity of GFP expression is displayed.

We wished to identify plant genes responding to infection by a close relative of TYLCV, *Tomato yellow leaf curl Sardinia virus* TYLCSV, and to analyze their function. To achieve these goals, we have generated a *N. benthamiana* transgenic line, named 2IRGFP, which allows monitoring virus-induction of host genes and their silencing. 2IRGFP plants contain a green fluorescence protein gene (GFP) expression cassette flanked by two repeats of the TYLCSV intergenic region IR [41]. Uninfected 2IRGFP plants display a basal low level of GFP. During infection, the TYLCSV Rep protein specifically recognizes the IRs flanking the cassette, and initiates replication and strong expression of the GFP transgene (Figure 1a). Therefore, induction of GFP expression directly correlates with viral replication, allowing monitoring the development of infection in plant tissues in both space and time in a simple visual, reliable and non-invasive manner (Figure 1b) [41]. Since the evaluation and monitoring of the viral infection is extremely straight-forward, we have used 2IRGFP plants as a tool in combination with VIGS to identify host genes with an impact in viral pathogenicity.

Prior to the use of TRV-based VIGS for a reverse genetics screen in 2IRGFP plants, we have shown that co-infection of TYLCSV with TRV did not alter the pattern of TYLCSV-dependent over-expression of GFP, even though TYLCSV accumulation was slightly delayed in plants co-infected with TRV compared to control plants [41]. At least three different proteins encoded by TYLCSV have been described to function as suppressors of gene silencing [42]. The possibility that a TYLCSV suppressor of gene silencing could counteract TRV-mediated silencing in TYLCSV-TRV co-infected plants was tested using either the endogenous *Sulfur* (*Sul*) gene (in 2IRGFP *N. benthamiana* plants) or a *GFP* transgene. The results indicated that co-infection with TYLCSV did not significantly alter the silencing phenotypes, confirming that TRV-mediated VIGS can be reliably used in combination with TYLCSV.

### 4.2. Selection and screening of candidate genes involved in TYLCSV infection

**Figure 2:**
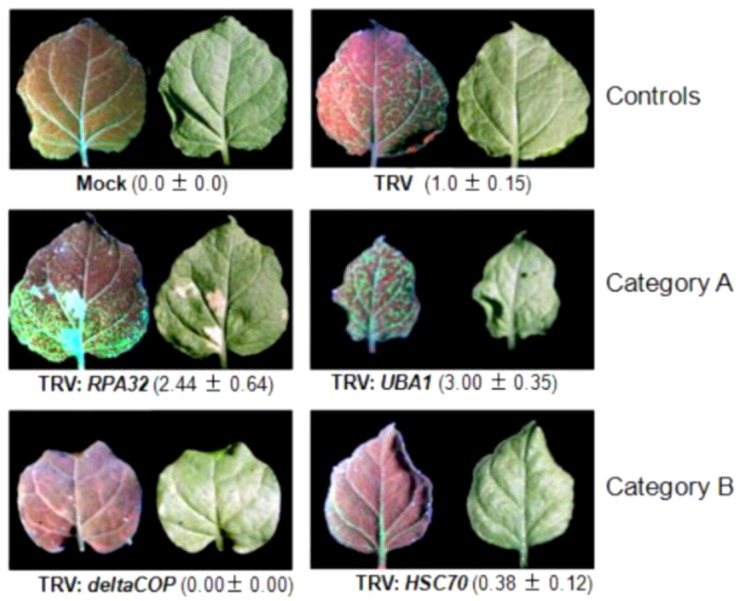
Screening of candidate genes in 2IRGFP transgenic *N. benthamiana* plants. Plants were co-inoculated with a TRV:*Gene* construct and TYLCSV. GFP expression was monitored daily up to 15 days post-inoculation (dpi). The picture shows GFP expression in one of the apical leaves under UV (left) and visible light (right) of 2IRGFP *N. benthamiana* transgenic plants 15 days after they were co-infected with TYLCSV and TRV constructs to induced silencing of genes classified in category A (Replication associated protein A, *RPA32*, and Ubiquitin activating enzyme 1, *UBA1*) or category B (Coatomer delta subunit, *deltaCOP,* and Heat shock cognate 70, *HSC70*). Leaves from control 2IRGFP plants are shown: agroinfiltrated with an empty binary vector (Mock) or with the empty TRV vector (TRV). The relative amount of TYLCSV DNA accumulated in co-infected plants was quantified by qPCR; results are shown below the images. Values are the mean of five to ten plants. The numbers correspond to the mean ±standard error. This experiment was repeated three times with similar results.

Genes potentially involved in TYLCSV infection was established following a literature search according to one of the four criteria: 1) they encode proteins binding geminiviral proteins; 2) they are exclusively or preferentially expressed in phloem tissues, to which TYLCSV is restricted; 3) they are trans-activated by C2 from the begomoviruses *Mungbean yellow mosaic virus* (MYMV) or ACMV [43]; 4) they are involved in cellular processes potentially required for geminivirus infection. A list of 114 genes was established. Since these genes belong to different plant species (the genome sequence of *N. benthamiana* was not available at the time), we performed homology analyses to identify sequence stretches conserved in diverse plant species, including *Arabidopsis* and tomato, which could serve as silencing targets. These sequences were used to design potentially efficient silencing siRNA molecules (Invitrogen Block-iT^TM ^RNAi designer tool). The fragments we chose for TRV-mediated silencing were those containing the largest number of potential siRNAs. Fifty-four of the initially selected 114 candidate genes fitted these pre-conditions; 37 were tested for their potential impact on TYLCSV infection upon silencing. The silencing recombinant TRVs were induced in 2IRGFP *N. benthamiana* plants, which were subsequently infected with TYLCSV. GFP over-expression was monitored daily from 9 to 15 days post-infection (dpi) under UV light; pictures and tissue samples were taken at 15 dpi (Figure 2). TYLCSV co-infection with empty TRV vectors or *Sul*-silencing TRV was used as control.

The effect of silencing the 37 host genes TYLVSV infection was classified into three categories: A- silencing of 7 resulted in an earlier or enhanced infection; B- silencing of 11 delayed, reduced or completely abolished infection; C- silencing of 19 did not induce a noticeable change in the pattern of infection. The identity and associated GO terms (biological process, cellular component and molecular function) for each of these genes are listed in Table 1. The genes identified in this screen can be classified into three functional groups discussed in more detail below: 1) genes with a previously known function in geminivirus infection; 2) genes involved in stress responses; 3) genes involved in posttranslational modifications. 

**Table 1:** List of genes whose silencing enhances (category A) or delays (category B) TYLCSV infection. The criterion for selection is indicated in each case. The accession numbers (ACC) of the homologous *Arabidopsis* gene used in the VIGS experiments are indicated. ND: not determined.

Identity	ACC *A. thaliana*	GO Biological process	GO Cellular component	GO Molecular function	Selection criteria
**Category A **					
Bearskin 2 (*BRN2*)	AT4G10350	Multicellular organismal development, positive regulation of gene expression, positive regulation of transcription, DNA-dependent, regulation of transcription, root cap development, secondary cell wall biogenesis	ND	Sequence-specific DNA binding transcription factor activity	Phloem over-expression
Importin alpha isoform 4 (*IMPA-4*)	AT1G09270	Host response to induction by symbiont of tumor, nodule or growth in host, protein transport, symbiont intracellular protein transport in host	Cytosol, host cell, intracellular	Protein binding, protein transporter activity	Interaction with CP
Lactoylglutathione lyase (*GLO1*)	AT1G15380	Carbohydrate metabolic process	ND	Lactoylglutathione lyase activity	Interaction with C3
Replication protein A32 (*RPA32/RPA2*)	AT3G02920	Unknown	ND	Nucleic acid binding	Interaction with Rep
Dehydration responsive 21 (*RD21*)	AT1G47128	Metabolic process, response to water deprivation	Apoplast, chloroplast, plasmodesma, vacuole	Cysteine-type endopeptidase activity, protein binding	Interaction with V2
RING-type E3 ubiquitin ligase (*RHF2A*)	AT5G22000	Megagametogenesis, microgametogenesis, proteolysis involved in cellular protein catabolic process, regulation of cell cycle	Plasma membrane	Zinc ion binding	Transactived by TrAP/C2
Ubiquitin activating enzyme (*UBA1*)	AT2G30110	Metabolic process, protein ubiquitination, response to cadmium ion, response to other organism, ubiquitin-dependent protein catabolic process	Cytosol, plasma membrane, plasmodesma	Ubiquitin activating enzyme activity, ubiquitin-protein ligase activity	Interaction with TrAP/C2
**Category B**					
4-coumarate:CoA ligase (*AT4CL1*)	AT1G51680	Metabolic process, phenylpropanoid metabolic process, response to UV, response to fungus, response to wounding	Unknown	4-coumarate-CoA ligase activity	Phloem over-expression
Allene oxide cyclase (*AOC1*)	AT3G25760	Jasmonic acid biosynthetic process, metabolic process, response to desiccation	Chloroplast, chloroplast envelope, chloroplast thylakoid membrane	Allene-oxide cyclase activity	Phloem over-expression
Barely any meristem 1 *(BAM1)*	AT5G65700	Anther development, floral organ development, gametophyte development, protein phosphorylation, regulation of meristem growth, regulation of meristem structural organization, trans-membrane receptor protein tyrosine kinase signaling pathway	Plasma membrane	Kinase activity, protein binding, protein self-association, protein serine/threonine kinase activity, receptor serine/threonine kinase binding	Interaction with C4
Coatomer delta subunit (*deltaCOP*)	AT5G05010	Intracellular protein transport, transport, vesicle-mediated transport	Cytosol, membrane, plasmodesma	ND	Interaction with C3
COP9 signalosome subunit 3 (*CSN3*)	AT5G14250	G2 phase of mitotic cell cycle, cullin deneddylation, photomorphogenesis	Cytosol, signalosome	Protein binding	Cellular process
Geminivirus Rep A-binding (*GRAB2*)	AT5G61430	Multicellular organismal development, regulation of transcription, DNA-dependent	Unknown	sequence-specific DNA binding transcription factor	Interaction with Rep
Heat shock protein cognate 70 (*HSC70*)	AT5G02500	Defense response to bacterium, defence response to fungus, negative regulation of seed germination, protein folding, response to cadmium ion, response to cold, response to heat, response to virus, stomatal closure	Apoplast, cell wall, chloroplast, cytoplasm, cytosol, membrane, nucleus, plasma membrane, plasmodesma	ATP binding, protease binding, protein binding	Phloem over-expression
Nuclear acetyltransferase (*NSI*)	AT1G32070	Pathogenesis, spread of virus in host	Chloroplast, nucleus	N-acetyltransferase activity	Interaction with NSP
Patatin-like protein 2 (*PLP2*)	AT2G26560	Cell death, cellular response to hypoxia, defence response to virus, lipid metabolic process, oxylipin biosynthetic process, plant-type hypersensitive response, response to cadmium ion	Cytoplasm, membrane	Lipase activity, nutrient reservoir activity	Phloem over-expression
Shaggy-related kinase kappa (*SK4-1/SKK)*	AT1G09840	Protein phosphorylation	Plasma membrane	ATP binding, protein serine/threonine kinase activity	Interaction with C4
SKP1-like 2 (*ASK2*)	AT5G08590	Phosphorylation, protein phosphorylation, response to osmotic stress, response to salt stress	Nucleus	Kinase activity, protein binding, protein kinase activity	Transactived by TrAP/C2

#### 4.2.1. Genes with a known function in geminivirus infection

Among the candidate genes that were found to exert an effect on TYLCSV infection when silenced, three have been implicated in begomovirus infection. 

***NSI*** (Nuclear shuttle interaction). *NSI* encodes a nuclear acetyl-transferase that physically interacts with the Nuclear shuttle protein (NSP) of CaLCuV. Over-expression of *NSI* resulted in enhanced infection [44], indicating that protein acetylation may coordinate replication of the viral genome with its export from the nucleus. This promoting effect of NSI on geminivirus infection is supported by our data, which showed that silencing of NSI negatively affects TYLCSV.

***GRAB2*** (Geminivirus Rep A-binding). *GRAB2* encodes a NAC-containing protein that interacts with *Wheat dwarf virus* (WDV) RepA in wheat [45]. Over-expression of *GRAB2* inhibits WDV replication in wheat cells. Unexpectedly, our results showed that low levels of GRAB2 enhanced TYLCSV infection. It is possible that GRAB2 has different roles in WDV and TYLCSV infections.

***RPA32*** (Replication protein A32). The gene product was shown to interact with the Rep protein of *Mungbean yellow mosaic India virus* (MYMIV) [46], repressing the Rep nicking and closing activities while promoting its ATPase activity. In our system, silencing of RPA32 resulted in enhanced TYLCSV infection. 

#### 4.2.2. Genes involved in stress responses

Five of the 18 genes identified in the screen as potentially involved in TYLCSV infection have been shown to play a role in plant stress responses. 

***HSC70-1*** (Heat shock protein cognate 70). HSC70-1 is one of five cytosolic members of the heat shock protein 70 family in *Arabidopsis* [47]. Infection with several plant viruses, including the geminivirus *Beet curly top virus* (BCTV), leads to enhanced expression of this gene family [48]. HSC70 interacts with the co-chaperone SGT1, which has been shown to be required for resistance against viruses [49]. The finding that silencing of *HSC70-1* results in impaired TYLCSV infection indicates that high levels of this protein are required for a successful geminivirus replication and spread. HSC70 may promote protein maturation during the virus multiplication cycle, and/or may be involved in virus cell-to-cell movement [50].

***RD21*** (Responsive to dehydration 21). RD21 is a cysteine protease. Tomato RD21 interacts with TYLCSV V2 in yeast (our unpublished results). Expression of *RD21* is induced following inoculation with *Botrytis cinerea* or *Pseudomonas syringae* (*Arabidopsis* eFP browser), or upon CaLCuV infection [51], pointing to a potential role in plant defense. Since silencing of *RD21* promotes TYLCSV infection, we hypothesize that this gene may also have anti-viral activities.

***PLP2*** (Patatin-like protein 2). PLP2 is a lipid acyl hydrolase, hydrolyzing membrane glycerolipids to produce monoacyl compounds and free fatty acids. Expression of *PLP2* is induced upon infection by CaLCuV [52]. Upon desiccation, *Arabidopsis* with a *plp2* mutation accumulates high levels of jasmonic acid (JA) [53]. Since in some cases activation of JA signaling negatively impacts geminivirus infection [54], over-production of JA due to *PLP2* silencing may explain the inhibition of TYLCSV infection.

***GLO1*** (Lactoylglutatione lyase). *GLO1* belongs to the glyoxalase system, which detoxifies methylglioxal (MG), a cytotoxic by-product of glycolysis [55]. Over-expression of *GLO1* results in increased tolerance to abiotic stresses [56]. Enhancement of the glyoxalase pathway in transgenic tobacco and rice helps maintaining low levels of reactive oxygen species (ROS) and MG [55]. Plant virus infection alters the expression of oxidative stress-related genes and induces oxidative stress correlated with the extent of symptoms [57]. In our system, silencing of *GLO1* could result in an increased accumulation of ROS, which would in turn favor viral infection.

***AOC1*** (Allene oxide cyclase 1). AOC1 catalyzes an essential step in the biosynthesis of jasmonic acid. Exogenous application of JA negatively impact geminivirus infection [54]. Therefore silencing of *AOC1*, which would presumably impair jasmonate biosynthesis, was expected to result in enhanced viral infection. Surprisingly, *AOC1*-silenced plants were more resistant to TYLCSV. It is possible that due to cross-talk between JA and salicylic acid (SA) signaling pathways, the silenced plants may accumulate high levels of SA, known to impair geminivirus infection [58].

#### 4.2.3. Genes involved in post-translational modifications (PTMs)

Strikingly, 8 of the 18 genes identified in the screen as involved in TYLCSV infection have been ascribed roles in post-translational modification (PTM) pathways: ubiquitination, rubylation, phosphorylation and acetylation. In this section, we will discuss the role of the four genes involved in ubiquitination since the involvement of this PTM in viral infections of plants and animals is well established. Ubiquitination consists in the addition of one (mono-ubiquitination) or more (poly-ubiquitination) ubiquitin moieties to a substrate protein; poly-ubiquitination generally results in the degradation of the modified protein by the 26S proteasome, while mono-ubiquitination can have other, non-fatal effects, such as changes in activity or sub-cellular localization [59]. In plants, ubiquitination contributes to multiple levels of defense [60], including resistance to viruses [61] and in plant-geminivirus interactions [54,62]. 

***UBA1*** (Ubiquiting-activating enzyme). UBA1 catalyzes the first step in ubiquitin conjugation. Interestingly, an *uba1* mutant in *Arabidopsis* can revert the constitutive defense response phenotype of *snc1*, which links UBA1 to plant defense [63]. We found that the tomato UBA1 interacts with TYLCSV C2 in yeast (unpublished results). Silencing of *UBA1* promotes TYLCSV infection, suggesting that a viral pathogenicity factor may suppress the activity of this enzyme. This hypothesis is in agreement with the previously described negative impact of C2 on ubiquitination [54,64], and would imply that C2 interferes with this process at multiple levels.

***RHF2A*** (RING-type E3 ubiquitin ligase). RHF2a links ubiquitin to target protein substrates. *RHF2a* is highly expressed in pollen, and to a lower extent, in vegetative tissues. This gene is up-regulated upon CaLCuV infection [4] and following *P. syringae* inoculation (Arabidopsis eFP browser). The potential role of *RHF2a* in plant responses to pathogens fits the findings that *RHF2a* silencing in the VIGS/2IRGFP system results in an enhancement of TYLCSV infection. 

**SCF** (Skp1/Cullin1/F-box protein). SCF is a multi-subunit E3 ligase. Its modular structure allows the incorporation of different substrate-binding subunits (F-box proteins) with more than 700 potential targets in *Arabidopsis* [65]. Interestingly, the C2 protein from several geminiviruses interferes with the SCF machinery [54,64]. In the VIGS/2IRGFP system we found two genes interacting with the SCF complex and involved in TYLCSV infection: *ASK2* and *CSN3*. *ASK2* belongs to a gene family encoding SKP1-like protein in *Arabidopsis*; it plays a role in cell division, development, and abiotic stress response *via* ABA signaling [66,67]. ASK2 interacts with GALA effectors from *Ralstonia solanacearum* [68] and with the VirF virulence factor from *Agrobacterium tumefaciens* [69], suggesting that ASK2 is a preferential target of pathogens virulence functions. Since V2 has been shown to trigger the degradation of the plant SGS3 in order to counter gene silencing [70], ASK2 may be essential for its efficient assembly into the SCF complexes, a process that may be assisted by C2 [54], ensuring the success of the viral infection. The finding that silencing of *ASK2* has a negative impact on TYLCSV in the VIGS/2IRGFP system is in line with this hypothesis.

**CSN3** (subunit of the de-rubylating CSN complex). CSN3 is one of eight subunits of the CSN (COP9 signalosome) complex, which de-rubylates CULLINs and therefore regulates the activity of CULLIN-based ubiquitin E3 ligases, including the SCF complex [71]. Geminivirus C2 was shown to interfere with the CSN de-rubylation activity, most likely through the interaction with CSN5 [64], presumably leading to an alteration of SCF-mediated ubiquitination. Since geminivirus infection is hindered in an *Arabidopsis csn5a* knock-down mutant [72], geminiviruses may be redirecting the activity of the CSN complex, taking over the regulation of SCF complexes rather than suppressing this process. Once again, this hypothesis is supported by the negative effect of *CSN3* silencing on TYLCSV infection. Taken together, the results obtained with *ASK2* and *CSN3* point at the usurpation of the SCF ubiquitination machinery by geminiviruses, involving different viral proteins and lines of attack. 

## 5. Identification of genes involved in resistance to TYLCV

### 5.1. Genes preferentially expressed in TYLCV-resistant tomatoes and the effect of their silencing on resistance

Breeding has allowed not only to develop TYLCV-resistant crops for farmers but the resistant plants have been the object of genetic studies aimed at understanding genes and signals involved in plant response to viruses [5]. To identify these genes, we have compared two inbred tomato lines issued from the same breeding program, which used *S. habrochaites* as a source of resistance: the TYLCV-susceptible line 906-4 and the TYLCV-resistant line 902, hereafter designated S and R, respectively [73]. Upon infection, plants from the S line present the typical disease symptoms of stunting, leaf yellowing and curling, contain large amounts of virus and produce a small number of fruits. In comparison, plants from the R line remain symptomless, yield, and contain several orders of magnitude less virus that S plants. 

We have postulated that resistance is sustained by a gene network responding to biochemical triggers induced by virus infection. In addition, we assumed that these genes are preferentially expressed in the R line and that their silencing will lead to the collapse of resistance. Comparing cDNA libraries from R and S plants, before and after infection, allowed the identification of about 70 genes preferentially expressed in R plants. Some of these genes were silenced using the TRV VIGS system. Fragments of 150 to 200 bp of the target genes were cloned in the TRVII vector. The TRVI and recombinant TRVII vectors were delivered to R and S tomato plants by agroinoculation [14] at the day of planting (20 days after sowing). Seven days later, the expression of the target gene was inhibited and the RNAi signal was conspicuous in the plant leaves and remained high for the duration of the experiments. This was the time the plants were caged with viruliferous whiteflies for a three days inoculation period. The effect of silencing was appraised during the next 40 days. TRV expression had no effect on subsequent TYLCV infection, neither enhancing nor depressing the virus spread [5]. 

**Figure 3:**
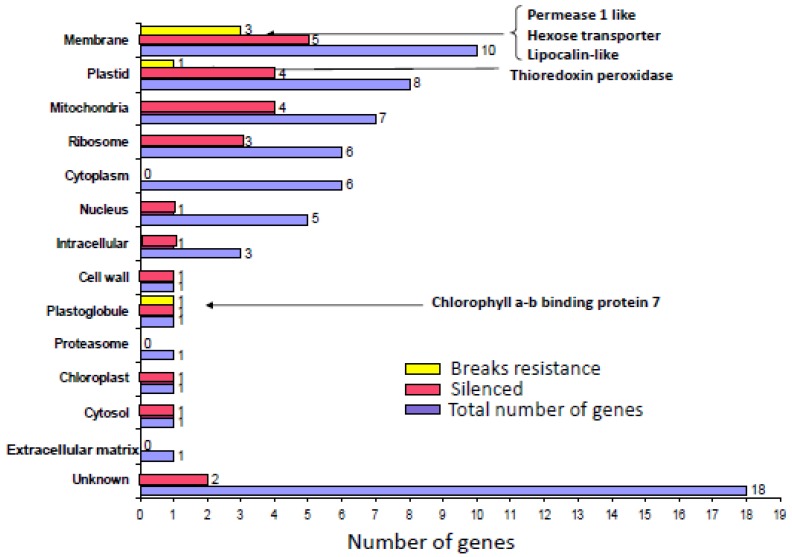
Genes preferentially expressed in R plants (Gene ontology, cellular component). The number of genes silenced so-far and the genes which silencing leads to collapse of resistance are indicated.

At present, we have silenced 25 out of the 69 genes preferentially expressed in R plants. Five genes out of the 25 tested led to the collapse of resistance when silenced (Figure 3). Hence, it seems that many genes are involved in the establishment of natural resistance to TYLCV. We summarize here the behavior of four genes preferentially expressed in R plants upon silencing and TYLCV inoculation. We also show that there seems to be a hierarchy in these genes.

**Figure 4:**
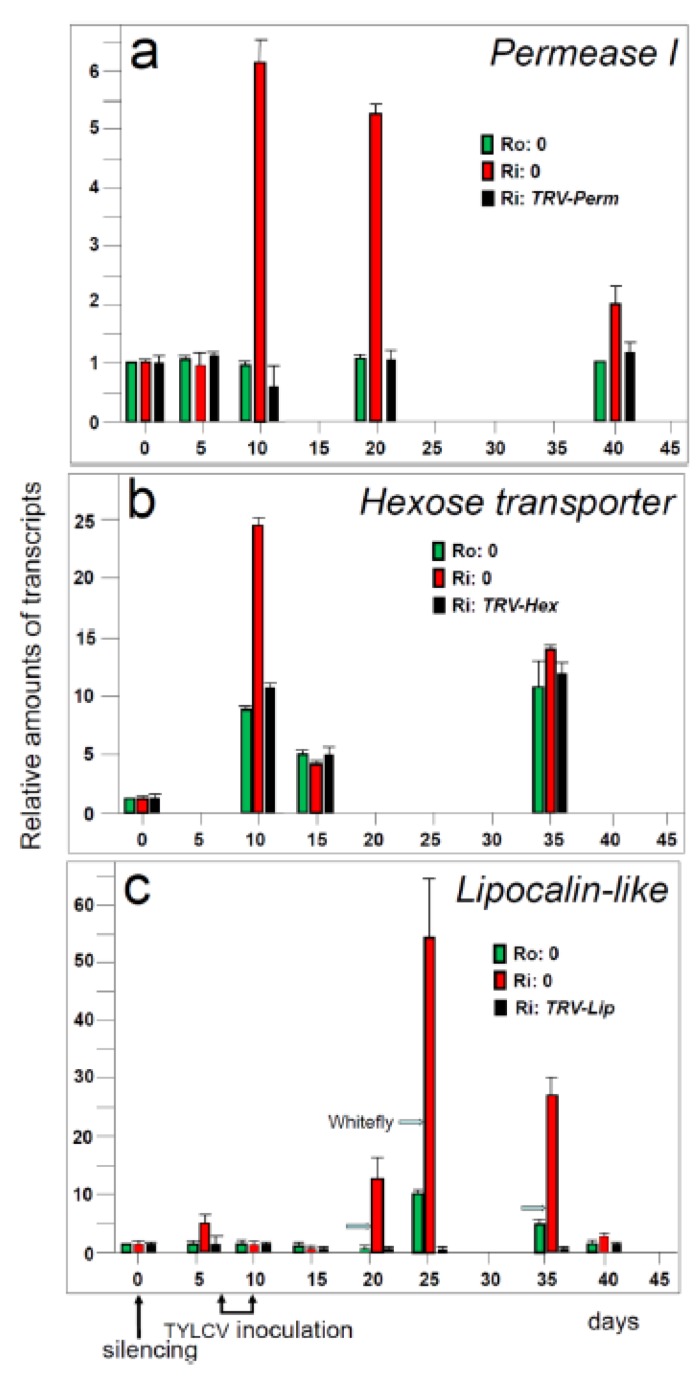
Relative amounts of transcripts of *Permease I*, *Hexose transporter LeHTe1*, and *Lipocalin-like* genes in R tomato plants (Ro:0), infected R tomato plants (Ri:0) and infected R tomato plants with silenced *Permease I* (Ri:*TRV-Perm*), *Hexose transporter LeHTe1* (Ri:*TRV-Hex*), and *Lipocalin-like* (Ri:*TRV-Lip*) genes. Tubulin RNA was used as a reference gene transcript for each of the plants analyzed by qPCR. The amount of transcript immediately before silencing (at day 0) is taken as 1. Average of triplicate measures of three different plants. Bars: standard error.

**Figure 5:**
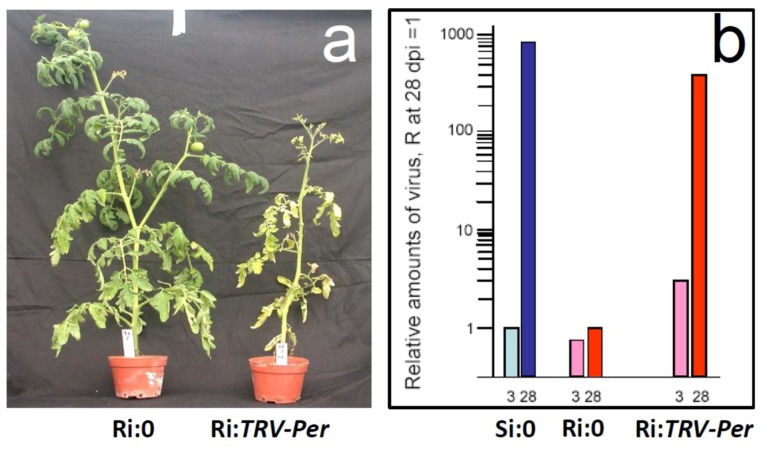
Collapse of resistance in infected R plants where the *Permease I* gene has been silenced. **a**: R tomato plants 8 weeks after TYLCV inoculation; Ri:0, not silenced; Ri:*TRV-Per*, silenced. Note that Ri:0 do not present symptoms and yield fruits, in comparison Ri:*TRV-Per* are symptomatic and present inhibited growth. **b**: Relative amounts of virus (measured by qPCR) in infected tomato plants 3 and 28 days after inoculation; Si:0 is S plants, Ri:0 is R plants and Ri:*TRV-Per* is R plants where the *Permease I* gene has been silenced. The amount of virus in Ri:0 plants at 28 dpi was considered as 1.

***Permease I****.* With the *PermeaseI-like* gene, we have shown for the first time that silencing a single gene can lead to the loss of TYLCV resistance in tomato plants. *Permease I-like protein* was preferentially expressed in non-inoculated R plants (compared to S plants) and was strongly up-regulated upon TYLCV inoculation [5]. Silencing this gene (Figure 4a) led to the collapse of the resistance phenotype: the R plants ceased to grow, developed typical yellowing and curling of leaves and contained amounts of virus similar to those measured in infected S plants (Figure 5). This permease may be involved in trafficking of macromolecules and signaling. 

***Hexose transporter LeHT1***. *LeHT1* is one of the three known tomato hexose transporter genes [74]. Plant hexose transporters are plasma membrane carriers, which function as proton/hexose symporters, mediating intercellular and long-distance transport of sugars [75]. They are involved in energy production [76], pathogenesis [77], pathogen defense [78] and programmed cell death (PCD) [79]. *LeHT1* is developmentally regulated, preferentially expressed in R tomato leaves, and upregulated upon TYLCV inoculation of R plants (Figure 4b). Infected *LeHT1*-silenced R plants ceased to growth and their leaves contained large amounts of virus in the vascular tissues and reduced sucrose concentrations [80], emphasizing the role of the hexose transporters and of sugars as part of a defense mechanism limiting virus movement. Necrosis appeared on the stem and petioles of the *LeHT1-*silenced R plants about three weeks after inoculation not only with TYLCV, but also with other viruses such as TMV and CMV [80]. Hence silencing of R plant *LeHT1* revealed a second line of defense associated with PCD features: DNA laddering, increased amounts of MAPKs, and release of reactive oxygen species (ROS) [80]. In most cases, PCD minimizes the pathogen spread [81], however, in the case of infection of *LeHT1-*silenced R plants, the plant defense mechanisms were unable to confine virus infection and the resistance collapsed. 

***Lipocalin-like protein*.** A gene encoding a putative lipocalin protein [82] with its typical barrel-shaped architecture [83], was expressed in the leaves of S and R tomatoes during a two week-long window, starting about 40 days after sowing (Figure 4c). This gene, coined *SlVSRLip*, was upregulated in R (but not S) plants upon infection but also, to a lesser extent, following whitefly feeding [82]. The association of lipocalins with virus infection has not been reported before. Following TYLCV inoculation, *SlVSRLip*-silenced R plants ceased to grow, developed disease symptoms, and contained large amounts of virus. As in the case of *LeHT1*, *SlVSRLip*-silenced R plants presented a PPCD-related necrotic response along the stems and petioles [82]. The role of *SlVSRLip* is not known, as it behaved differently than the known tomato lipocalins [83], which appear to protect plants from temperature-induced stresses [84]. 

***Pectin methylesterase*.** Another gene preferentially expressed in R plants was a *Pectin methylesterase*. This gene is a member of a large family encoding enzymes that modify plant cell wall pectins. Pectin methylesterases play a role in the plant host defenses against cold, wounding and phloem-feeders [85]. They have also been involved in virus-induced gene silencing [86] and in virus systemic infection [87]. Contrary to the three genes described above, silencing *Pectin methylesterase* did not affect the resistance of R plants. Hence, although *Pectin methylesterase* is more expressed in R than in S plants, this gene is probably not located at a bottleneck of the resistance network. Thus, not all the genes preferentially expressed in R plants play the same role in the establishment of resistance to TYLCV.

### 5.2. Hierarchy of genes involved in resistance to TYLCV

We hypothesized that the genes conferring resistance in R plants are organized in an interconnected hierarchical network. We therefore tested the hypothesis that the silencing of one gene will cause the down-regulation of genes downstream in the network. Expression of *SlVRSLip* was estimated in R plants in which *LeHT1* had been silenced [82]. In the *LeHT1*-silenced R plants, the expression of *SlVRSLip* was totally inhibited. Conversely, silencing of *SlVRSLip* did not affect the expression of *LeHT1*. Hence, *SlVRSLip* is downstream of *LeHT1* in the hierarchy of the resistance network [82]. Silencing a *Permease* gene did not affect the expression of either *SlVRSLip* or *LeHT1*; conversely, silencing either *SlVRSLip* or *LeHT1* did not affect the *Permease* gene expression, indicating that the later gene does not belong to the *LeHT1/SlVRSLip* branch of the network. *SlVRSLip* and *LeHT1* do not seem to be linked by any obvious biochemical or physiological link. However, as a consequence of *LeHT1-*silenced, the concentration of sucrose in leaves was lower of than that in non-silenced R tomatoes [82]. It has been already reported that silencing *LeHT* genes decreased hexose accumulation in tomato fruits by half [88]. Hence the inhibition of sugar transport due to *LeHT1* silencing resulted in a limited level of cellular sucrose, and consequently energy, to activate and maintain the resistance response [78]. Sugars act as secondary messengers [89] and sugar sensing mediates a direct link between carbohydrate metabolism and the defense response [78]. In this context, intracellular sugars may up-regulate the expression of *SlVSRLip* in R plants upon TYLCV infection, contributing to resistance by increasing lipocalin ROS scavenging. A reduction in the intracellular concentration of sugars due to *LeHT1* silencing may inhibit the signal-transduction pathway leading to the activation of *SlVSRLip*. 

## 6. Discussion

We have shown that TRV-VIGS is a tool of choice to discover plant genes responding to TYLCSV infection. Using the 2IRGFP *N. benthamiana* transgenic line, we have been able to demonstrate that silencing of 18 out of 37 analyzed host genes alters TYLCSV infection. An attractive feature of this screening method is the fact that candidate genes are tested in the context of the infection, hence the genes discovered are likely to be biologically relevant. On the other hand, we cannot rule out that some of the tested genes have not been efficiently silenced, rendering their potential impact on TYLCSV infection undetectable. A strategy similar to VIGS/2IRGFP is more difficult to apply to tomato, since expression of GFP in leaves does not bear green fluorescence. Therefore, the genes discovered in the *N. benthamiana* 2IRGFP plant screen could be validated subsequently in tomato.

It is striking that almost half the genes shown to interfere with TYLCSV infection are involved in processes related to PTMs, such as ubiquitination, rubylation, phosphorylation, acetylation or folding. It has been postulated that PTMs provide means to respond quickly to environmental stimuli in a fast and efficient way critical for the plant survival. Thus, it is not surprising that PTMs affect viral infection and may be preferred targets of viral pathogenicity factors. Increase evident obtained in the last years confirm the central role played by PTMs in virus-host interactions, being both manipulated by viruses to achieve a successful infection and used by the host as an important defense mechanism [59,61]. 

We have also applied the TRV-VIGS reverse genetics tool to discover genes involved in tomato natural resistance to TYLCV. The current view to plant responses to stress involve integrated transcriptional and cellular changes that result in physiological adaptations expressed as resistance in certain genotypes, which may be regulated by metabolite and hormone signaling pathways [90]. Accordingly, we have postulated that resistance to TYLCV is sustained by a gene network. Indeed, we have identified several genes from R plants which, when silenced, lead to the collapse of resistance. We found a beginning of hierarchy in the TYLCV-resistance network, where *SlVRSLip* is downstream of *LeHT1*. To uncover the genes up- and downstream *LeHT1* in the resistance network we are using a home-made oligonucleotide microarray to analyze the transcriptome re-programming in leaves of *LeHT1*-silenced R plants using a home-designed microarray [91]. Resistance to TYLCV may consist of several layers of defense - a general feature of the plant response to pathogens [38]. This multilayer response starts with a basal response and production of general pathogen-associated molecular pattern molecules (PAMPs), followed by activation of MAPK-signaling cascades and production of antimicrobial compounds [92]. The next layer of resistance usually involves the expression of genes related to the plants response to specific pathogens, in our case *LeHT1* and *SlVSRLip*. 

Since the R line's resistance to TYLCV was introgressed from *S. habrochaites*, it would be of interest to determine whether the genes that are preferentially expressed in R tomato plants were introgressed from this wild tomato species. It is worth noting that the three genes we have studied are located on three different chromosomes: *Permease I-like protein* is on chromosome 3, *LeHT1* is on chromosome 2, and *SlVSRLip* is on chromosome 10 (http://solgenomics.net accesed 18 February 2013). If these three genes originate from the wild *S. habrochaites* genitor, they must have been introgressed as three chromosomal fragments during breeding and selection for resistance. 

In summary, the results presented here are a good example of the potential of VIGS as a tool for functional studies in plant-virus interactions, providing at the same time new insights into the roles that specific plant genes play during geminivirus infection. In the genomic era, the completion of genome sequences of many important plant species, including *N. benthamiana* and tomato [93,94], together with the efforts made to improve the efficiency and applicability of the VIGS system to different hosts, are contributing to make this technology an essential tool for high-throughput functional genomics studies in plants.
